# Finger-Counting and Numerical Structure

**DOI:** 10.3389/fpsyg.2021.723492

**Published:** 2021-09-28

**Authors:** Karenleigh A. Overmann

**Affiliations:** Center for Cognitive Archaeology, University of Colorado Colorado Springs, Colorado Springs, CO, United States

**Keywords:** numbers, number system, finger-counting, cultural differences, external representation

## Abstract

Number systems differ cross-culturally in characteristics like how high counting extends and which number is used as a productive base. Some of this variability can be linked to the way the hand is used in counting. The linkage shows that devices like the hand used as external representations of number have the potential to influence numerical structure and organization, as well as aspects of numerical language. These matters suggest that cross-cultural variability may be, at least in part, a matter of whether devices are used in counting, which ones are used, and how they are used.

## Introduction

Number systems differ cross-culturally in their content, structure, and organization. For example, in Amazonian Brazil, Desana counting goes up to “twenty,” and numbers are grouped into four groups of five based on the hands and feet (Silva, 2012). The names of these numbers are long phrases describing combinations of fingers and toes. Their length means they are not easily recited in sequence, and the Desana are not known to use them that way in counting. In Papua New Guinea, Oksapmin counting uses the body as a tally, the numbers are sequentially named according to the associated body parts, and they are related to each other ordinally, by their order within the sequence; as such, they lack the relations needed for Western-style addition and subtraction (Saxe, 2012). In Africa, Yoruba counting uses cowrie shells organized into groups and bundles. Numbers are dynamic arrays, so the number 600 can be expressed in a well-formed fashion as “50 in 12 places, 60 in ten places, or 100 in 6 places,” with no particular form considered better or more correct than any other (Jeffreys, 1948, 48). In the Pacific, Polynesians counted by sorting every tenth item to create piles that meant tens, then hundreds, and then thousands, and they counted with singles, pairs, and fours, creating exponential structure and different sequences for counting different types of objects (Overmann, 2020).

Historically, the reason(s) why cultural number systems vary in their structure and organization has been unclear, especially for models seeking answers within the brain. Here a simple explanation is offered: Variability may be at least partly a matter of whether devices are used, which ones are used, and how they are used. Any particular social group using a particular device for counting has opportunities to make different decisions about how a device is used, potentially creating different outcomes in numerical structure and organization. How variability in use informs numerical structure and organization can be illustrated with the hand, a device commonly used for counting that is physically and neurally identical across cultures.

## Counting on the Hand(S)

Finger-counting in any form is assumed to represent a common sensorimotor mechanism for representing and processing numerical information, one leveraging the neurological interaction between the parts of the brain that appreciate quantity, “know” the fingers, and plan movements (Marinthe et al., 2001; Roux et al., 2003; Vandervert, 2017). Sensorimotor commonality with expressive variability suggests the way the hand is used in counting is not neurally predetermined, but rather, culturally inflected (e.g., Bender and Beller, 2011, 2012). Use patterns may ultimately be tracible to social needs and/or ecological conditions, a historical aspect of use beyond the present scope, which focuses on structural and organizational outcomes.

### Restricted Number Systems

**FIGURE 1:**
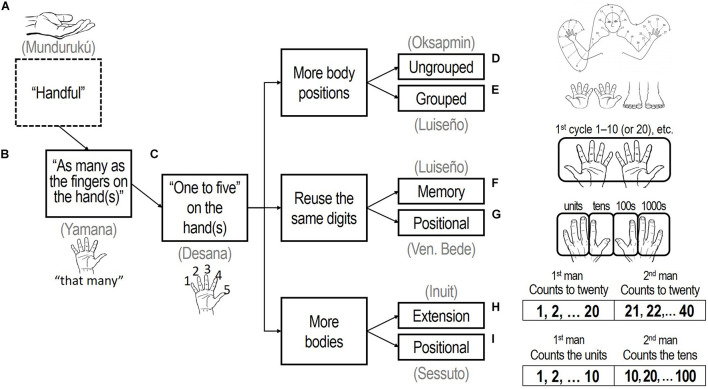
How the hand is used in counting affects numerical structure and organization. Key: **(A)** a handful; **(B)** without counting the fingers; **(C)** with the fingers counted; **(D)** adding other body parts; **(E)** adding the feet; **(F)** remembering additional cycles; **(G)** using the fingers to track cycles; **(H)** sequential collaboration; and **(I)** exponential collaboration. Oksapmin body-counting panel **(D)** is adapted from Saxe (2012), Figure 12, p. 46; images of hands and feet are from the public domain.

The Mundurukú (Figure 1A) are indigenes of Amazonian Brazil whose counting ranges from “one” to “about four” (Rooryck et al., 2017). Above “about four,” Mundurukú use the idea of a handful (Pica and Lecomte, 2008). This seemingly involves the hand not so much for the quantity of its fingers, but rather, for what it can grasp, potentially collapsing any distinction between “how many” and “how much” and perhaps grounding numerical concepts in manuovisual experience, as demonstrated by improvements in the ability to appreciate quantity when viewed objects are sized to be grasped (Ranzini et al., 2011).

The hand can also be used without counting the fingers (Figure 1B), creating phrases typically glossed “as many as the fingers on my hand.” This was observed for the Yamana of Tierra del Fuego, whose words for “five” and “ten” were *yëkýli yéš*, “one hand,” and *kompéi yéš*, “two hands” (Gusinde, 1931, 1838). The fingers can also be enumerated individually (Figure 1C), as was noted for the Desana of Amazonian Brazil, whose terms for “five” and “ten,” *yuhuru mõhõtõ* (“one hand”) and *pẽmõhõtõ* (“two hands”), are accompanied by companion terms for the remaining fingers; the term for “six,” for example, is *yuhuru mõhõtõ yuhuru nĩã\~texthooktopã*, “one hand, one from other hand” (Silva, 2012, 183).

### Counting Beyond “Ten” and Unrestricted Number Systems

Numbers higher than “ten” can be counted by continuing from the hand to other parts of the same body, using it like an ungrouped tally. For example, Oksapmin body-counting (Figure 1D) counts to 27 by sequentially moving from the fingers of the right hand up the right arm, across the head, and down the left arm before finishing with the left fingers (Saxe, 2012).

Numbers higher than “five” or “ten” can also be counted with the feet (Figure 1E). For example, the Luiseño of California counted “chiefly by means of the fingers and toes” (Sparkman, 1905, 657). This method often creates groupings by fives and twenties: “ten,” “all my-hand finished”; “fifteen,” “all my-hand finished and one my-foot”; “twenty,” “another finished my-foot the-side” (Dixon and Kroeber, 1907, 689).

The hands of the same person, with or without the feet, can also be used to count additional cycles of “ten” or “twenty.” This requires keeping track of the number of cycles in some fashion. Formations like the Luiseño self-reference suggest the counter simply remembers the number of cycles (Figure 1F). Luiseño numbers also show that while some possibilities are mutually exclusive—counting cannot be both grouped and ungrouped, for example—others are not, since self-reference (“my hand”) can be combined with counting only the hands or both hands and feet.

Particular fingers can be used for keeping track of the tens, hundreds, and thousands (Figure 1G), as was the case with the finger-counting system recorded by the Venerable Bede in 725 CE (Nishiyama, 2013). Units were displayed with the little, ring, and middle fingers of the left hand, tens with the left index finger and thumb. The right index finger and thumb displayed the hundreds, while the right little, ring, and middle fingers tracked the thousands. Numbers in the range 10,000–90,000 were indicated by touching various parts of the body with left hand, 100,000–900,000 with the right, while 1,000,000 was indicated by clasping the hands together with interlocked fingers (Richardson, 1916).

### Collaborative Counting

Multiple people can collaborate in counting. One technique adds the hands (and toes) of additional people to extend the range of counting (Figure 1H). Inuit, a people of the Arctic regions of Canada, Greenland, and Alaska, counted in this manner, with “twenty-one,” *inûp áipagssâne atausek*, meaning “one [finger] on the second man”; “thirty-eight,” *inûp áipagssâne arfinek pingasut*, “three toes on the second man’s second foot”; and “forty,” *inûp áipagssâ nâvdlugo*, “the whole [digits] of the second man”(Nansen, 1893, 194–95).

Another collaborative technique divides the responsibility for keeping track of the units, tens, and hundreds between multiple people (Figure 1I). Examples are found in Africa and Oceania (Schrumpf, 1862; Collocott, 1927). In this type of counting, the first man “counts the units on his fingers by raising one finger after the other and pointing out the object counted or, if possible, touching it. The second man raises a finger … for every ten, as soon as it is completed. The third man counts the hundreds”’ (Cajori, 1899, 35).

### Other Variability

The variability described above adds to the differences in structure and organization created by using the fingers, the spaces between them, or the segments, joints, or tips; whether or not the thumb is included; and whether the fingers are seen as additive or subtractive. These combinations influence productive grouping in amounts that range from “four” “to “twenty,” as well as verbal expressions for “eight” that add “three” to “five” or subtract “two” from “ten.” Which fingers are used to initiate and finish the counting sequence and the manner of progressing between the two can influence structure as well, since the fingers can be used sequentially on successive hands, or symmetrically and simultaneously, with, for example, three fingers on each hand displayed to mean “six” (Tempels, 1938). In some cases, the meaning of the verbal expression may be unclear and can be understood only in conjunction with the accompanying gesture (Olderogge, 1982). For example, for the Bashila of Zaire, the phrase for “seven,” *t∫inε lubali*, means “four on one side”; this is accompanied by a counting gesture that shows four fingers on one hand and three on the other (Tempels, 1938). Other decisions with perhaps less influence on numerical structure include which hand to start with; whether to use anatomic or spatial symmetry; whether fingers are extended, bent, tapped, or shaken; and when counting shifts between hands.

## Other Effects of Using the Hand

Using the hand as a material device for counting influences properties like linearity, sequentiality, and discreteness (Gelman and Gallistel, 1978). Linearity and sequentiality are functions of the topographical layout of the portions of the brain that appreciate quantity (Harvey et al., 2013, 2015) and control hand sensation and movement (Penfield and Rasmussen, 1950; Penfield and Jasper, 1954), plus the visual experience of the hand as a physical device, plus the fact that finger-counting requires less attention and is more reliable if the hand is used the same way every time (Overmann, 2018). As for discreteness, the upper end of the subitizing range is fuzzy: “about three or four.” However, it is difficult for this to remain fuzzy once the hand becomes involved in counting; “three” and “four” become discrete when represented on adjacent fingers (Overmann, 2018).

Language for numbers is also influenced by finger-counting, not just in properties like productive grouping and extent, but also in the form that verbal expressions, if used, might take. For example, in Africa, displaying numbers with the fingers need not be accompanied by any spoken expression (Lichtenstein, 1812), or a numerical gesture might be accompanied by a verbal expression that merely draws attention to the hand (Olderogge, 1982). Verbal expressions might gloss the fingers as they are counted with unindividuated expressions essentially meaning “and another.” In the city of Gobabis in eastern Namibia, the term *neba hawu* (“this one is swallowed”) was reported for the numbers “seven” through “ten” as the four right fingers were sequentially indicated (Olderogge, 1982).

Verbal expressions might describe particular configurations of the fingers and toes in detail, creating lengthy and cumbersome phrases, as was noted earlier for Desana numbers. This might influence the silent or accompanying use of the hand as preferable to sequential recitation. Displaying the fingers plausibly influences and supports the ultimate truncation of lengthy verbal expressions to short, conventional syllables, since providing the exact meaning visually enables a phrase meaning “one hand, [and] one from other hand” (Silva, 2012, 183) to omit shared components. For example, the Bashila phrase for “nine,” *pabula kimɔ kia likumi*, can be shortened to *pabula* (Tempels, 1938, 52).

Collaborative counting involves sequential recitation. During this process, the names for numbers are simplified as different individuals keep track of units, tens, and hundreds, all of which involve counting only from “one” to “ten.” Such counting may be performed socially. For example, Tongan collaborative counting was a ceremony conducted to prepare for public feasting and commodity distribution (Collocott, 1927). Exposure to performed numerical naming may bear on lexicalization, the ability to generate new number phrases from a small lexicon of number-words. In a decimal system, these typically include “one” through “ten” and multiples of “ten” like “hundred” and “thousand.” Cross-linguistically, lexicalization appears to be a secondary phenomenon, as words for small numbers (which emerge first in any number system) tend toward irregularity, with lexicalized expressions for higher numbers (which emerge later in any number system) being generated in a regularized manner. For languages with unlexicalized numbers (e.g., the Desana), hearing the names recited in sequence might influence its emergence.

## Conclusion

Historically, the brain has been viewed as the locus of conceptualization. On this account, brains conceive numbers, with variability of structure and organization indicating the range of things the brain can potentially do, though why it should do things differently in some circumstances but not others has been difficult to explain, particular in that not all societies have numbers (Hurford, 1987; Chomsky, 2004). At some point, concepts may be externalized onto material forms like tallies and notations for reasons that are also unclear, with devices being considered passive recipients of that mental content.

Another approach starts with the visual experience of quantity and symbolic notations, recognizing both as involving material forms that are engaged manuovisually. In this view, numerical conceptualization starts with the perceptual ability to appreciate quantity and a world of appreciable quantity whose material substance can be altered in ways that bring forth meaning (Malafouris, 2010). Bridging the gulf between perceptual experience and symbolic notations are devices like fingers and tallies that are also engaged manuovisually to represent and manipulate number (Overmann, 2018). In this model, rather than being the passive recipients of mental content, external representations have a constitutive role. For example, symbolic notations are understood as structuring and organizing numerical concepts in a way that informs how numbers are acquired and what they are understood to be (Schlimm, 2018); this same role is reasonably construed for precursor devices like fingers. Variability in structure and organization then becomes at least partially a matter of whether and how devices are leveraged to represent and manipulate numerical information. Fingers, in this case, provide a visible device, albeit one neurally linked to quantity perception, whose form and manipulation inform basic structural and organization properties of number systems.

The use of the hand as a material device for representing and manipulating numbers is a source of variability in numerical structure and organization. Recognizing this opens up the possibility that material devices like cowrie shells and behaviors like sorting might influence numerical structure and organization as well.

## Author Contributions

KAO was responsible for all of the research, analysis, and writing.

## Conflict of Interest

The author declares that the research was conducted in the absence of any commercial or financial relationships that could be construed as a potential conflict of interest.

## Publisher’s Note

All claims expressed in this article are solely those of the authors and do not necessarily represent those of their affiliated organizations, or those of the publisher, the editors and the reviewers. Any product that may be evaluated in this article, or claim that may be made by its manufacturer, is not guaranteed or endorsed by the publisher.
